# Epigenetics in Cancer: A Hematological Perspective

**DOI:** 10.1371/journal.pgen.1006193

**Published:** 2016-10-10

**Authors:** Maximilian Stahl, Nathan Kohrman, Steven D. Gore, Tae Kon Kim, Amer M. Zeidan, Thomas Prebet

**Affiliations:** 1 Department of Internal Medicine, Section of Hematology, Yale School of Medicine, New Haven, Connecticut, United States of America; 2 Department of Internal Medicine, Section of Hematology, Yale Cancer Center at Yale University, New Haven, Connecticut, United States of America; Brigham and Women's Hospital, UNITED STATES

## Abstract

For several decades, we have known that epigenetic regulation is disrupted in cancer. Recently, an increasing body of data suggests epigenetics might be an intersection of current cancer research trends: next generation sequencing, immunology, metabolomics, and cell aging. The new emphasis on epigenetics is also related to the increasing production of drugs capable of interfering with epigenetic mechanisms and able to trigger clinical responses in even advanced phase patients. In this review, we will use myeloid malignancies as proof of concept examples of how epigenetic mechanisms can trigger or promote oncogenesis. We will also show how epigenetic mechanisms are related to genetic aberrations, and how they affect other systems, like immune response. Finally, we will show how we can try to influence the fate of cancer cells with epigenetic therapy.

## Introduction

Over the past two decades, the connection between cancer and epigenetic regulation has been a promising venue for research. From the first evidence of the epigenetic silencing of tumor suppressor genes’ promotors, we now have a more complex and multidimensional picture, integrating several layers of (dys)regulated DNA methylation, histone modification, and micro RNA modulation. Maybe most important, epigenetic regulation has emerged as an intersection of several key hallmarks of cancer such as immunology, metabolism, or aging [1,2].

Many of these discoveries were initially described in context of hematological malignancies, and, acknowledging significant exceptions, their counterparts in solid tumors have not been so easy to demonstrate. Similarly, the benefit of epigenetic targeting has been identified in myelodysplastic syndromes and acute myeloid leukemias with the use of DNA hypomethylating agents and, to a lesser extent, histone deacetylase inhibitors. As our tools to study epigenetics progressed, our arsenal of epigenetic-targeted drugs started to expand. Hematology is at the cutting edge of research on the development of drugs targeting epigenetic regulators, including DOT1L, BET proteins, LSD1, and IDH1/2 inhibitors.

In this review, we will present the current trends in epigenetic research encompassing the biology of epigenetics, interactions with other cancer mechanisms, and drug development. Research on myeloid malignancies will be used to illustrate these different topics.

## Mechanism of Epigenetics

**Fig 1:**
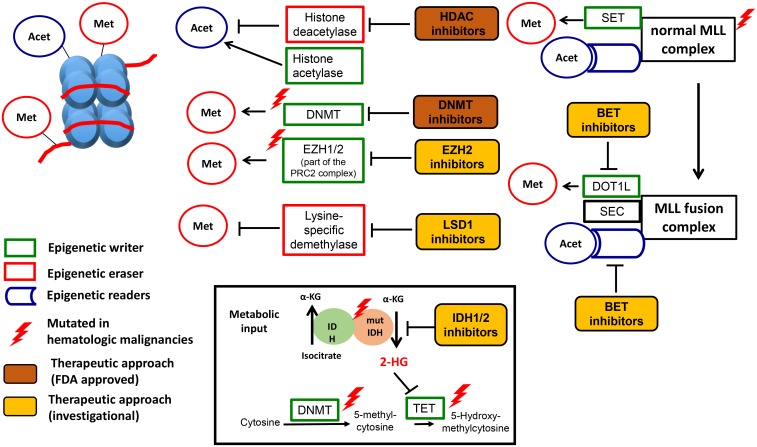
Epigenetics in hematological malignancies. **Epigenetic regulation, dysregulation and therapeutic targets.** DNA (red) forms a complex with histone proteins (light blue) to form nucleosomes. Each nucleosome consists of DNA wrapped around a unit of eight histone proteins. Epigenetic regulation: Epigenetic marks are placed both on the DNA and histones by epigenetic writers (in green). DNMT3A, TET2, EZH1/2, and the histone acetylase are examples of epigenetic writers. EZH2 is part of the Polycomb Repressive Complex 2 (PRC2), which also contains ASXL1, EED, SUZ12, and RBAP48. Epigenetic marks are removed by epigenetic erasers (in red), e.g., Lysine specific demythylases (LSD) and histone deacetylases (HDAC). IDH1/2 provide metabolic input by providing a-KG, which is an important substrate for the catalytic domain of other epigenetic regulators like TET2. Finally, epigenetic marks are recognized by epigenetic regulators though special reader domains (in blue), which lead to the recruitment of epigenetic regulators to DNA and histones. Examples of reader domains are the plant homeodomain (PHD) finger proteins and the bromodomain and extraterminal (BET) family of proteins. The BET family has four members, including bromodomain-containing proteins 2, 3, and 4 (*BRD2*, *BRD3*, and *BRD4*) and *BRDT*. The wild-type mixed-lineage leukemia (*MLL*) gene is post-translationally cleaved into N-terminal and C-terminal fragments that re-associate to form the MLL complex. The C-terminal fragment contains a SET domain, which methylates H3K4, and the N-terminal fragment contains PHD fingers and a bromodomain, which serve as reader domains. Epigenetic dysregulation: The complex epigenetic regulatory program is disturbed in hematologic malignancies by mutations in epigenetic regulators (indicated by red thunderbolt) or by the recruitment of large multi-protein complexes like the MLL fusion complex (purple circle). Translocations involving the MLL gene account for the vast majority of infantile and approximately 10% of adult leukemias. Following translocation, a fragment of the N-terminal portion of MLL is fused in frame with a translocation partner, leading to the formation of novel MLL-fusion protein complexes, including the super elongation complex (SEC) and the DOT1-Like Histone H3K79 Methyltransferase (DOT1L) complex. The DOT1L complex leads to misdirected H3K79 methylation, which has been shown to sustain the expression of key pro-leukemic genes such as the *HOXA* genes and *MEIS1*. The SEC complex phosphorylates RNA polymerase II (POL II) facilitating its recruitment to the promoters of crucial oncogenes such as *MYC*, *BCL2*, and *CDK6*. Metabolic dysregulation is caused by IDH1/2 mutations, which leads to the production of an abnormal metabolite in the cell, 2-hydroxyglutarate (2HG), which can inhibit the hydroxylation of 5-mC by TET2. Therapeutic targeting: Food and Drug Administration (FDA)-approved are DNMT3A inhibitors for AML and MDS and HDAC inhibitors for T cell lymphoma and multiple myeloma, respectively (in orange). Several investigational drugs (in yellow) are in different stages of preclinical and clinical development. Adopted from Semin Hematol. 2015 Jul;52(3):172–83 [8]

Epigenetics is defined as heritable changes in gene expression that are not due to any alteration in the DNA sequence [3,4]. Epigenetic modifications are placed by epigenetic writers and removed by erasers in a dynamic but highly regulated manner [5]. Many different DNA and histone modifications have been identified to determine the epigenetic landscape (Fig 1) [6–8].

The DNA (5-cytosine)-methytransferases (DNMTs) add methyl groups to cytosine in CpG dinucleotides in DNA and the TET family of proteins catalyze 5-methylcytosine to 5-hydroxymethylcytosine [9–11].

Histone acetylation is associated with elevated transcription, while deacetylated histones are associated with gene repression. Acetylation removes the positive charge on the histones, which leads to a decrease in the interaction of the N termini of histones with the negatively charged phosphate groups of DNA. Subsequently condensed chromatin (heterochromatin) is transformed into a more relaxed structure (euchromatin), which leads to increased levels of gene transcription [6]. Histone acetylases and histone deacetylases (HDACs) add and remove acetyl groups from histones and are critical regulators of gene expression [12].

Methyltransferase Enhancer of Zeste Homologue 2 (EZH2) is an integral part of the polycomb repressive complex 2 (PRC2), which maintains transcriptional silencing through posttranslational histone modifications [11]. Transcriptional silencing is initiated by recruitment of PRC2, which, through EZH2, induces mono-,di-, and trimethylation of lysine 27 of histone H3 (H3K27). PRC1 recognizes H3K27me3 and mediates ubiquitylation (Ub) of lysine 119 of histone H2A (H2AK119), which is thought to lead to the recruitment of DNMTs to target loci and silencing of gene expression (Fig 1) [13].

Lastly, metabolic input is mediated by the isocitrate dehydrogenase enzyme (IDH), which catalyzes the conversion of isocitrate to alpha-ketogluatarate (α-KG) [14]. Dioxygenase enzymes, which include the TET family of enzymes and the Jumonji –C domain-containing (JMJC) family of histone lysine demethylases, are α-KG dependent enzymes (Fig 1) [15].

The application of new molecular techniques, namely, next generation sequencing (NGS) coupled with DNA methylation profiling as well as chromatin immunoprecipitation (ChIP-Seq) [16–18] and epigenome editing technology based on CRISPR-Cas9 approaches [19,20], allowed researchers to characterize the impact of epigenetic modification not only on promotors but on the entire genome [21]. Typical patterns of histone modifications exhibited at promoters and regulatory domains (insulators, enhancers, repressors) have been identified [22]. Many chromatin regulators also survey the epigenetic landscape using specialized domains to dock at specific domains within the genome, leading to recruitment of functional complexes regulating DNA transcription [5,23]. Many “writers” and “erasers” possess this chromatin “reader” ability in addition to their catalytic activity and respond to information conveyed by upstream signaling cascades. These regulators use complex three-dimensional binding pockets (e.g., bromodomain, PHD finger), which allow readers with similar binding domains to dock at different modified residues or at the same amino acid displaying a different modification state (Fig 1) [24]. The multifaceted mechanism that chromatin readers use to decipher the epigenetic landscape is exemplified by the fact that many readers have more than one reader domain, and binding to chromatin is influenced by neighboring histone modifications [5,25]. Single-cell epigenetic profiling will further promote our understanding of epigenetic regulation by addressing the issue of epigenetic heterogeneity of cancer [26].

## Role in Cancer

Epigenetic dysregulation manifests in cancer with global DNA hypomethylation, causing genomic instability as well as silencing of specific tumor suppressor genes and of microRNA (miRNA) genes by hypermethylation [4,27–31].

Large-scale studies of DNA methylation have detected extensive hypomethylated genomic regions in gene-poor areas in cancer cells and demonstrated that the degree of hypomethylation of genomic DNA increases as the tumor progresses from a benign proliferation of cells to an invasive cancer [32,33].

Global hypomethylation promotes tumorigenesis by the generation of chromosomal instability (promoting chromosomal deletions and rearrangements), reactivation of transposable elements (further disrupting the genome), and loss of imprinting [34–39]. Furthermore, gene inactivation through hypermethylation of the CpG islands in the promoter region has been identified for many tumor suppressor genes, including the retinoblastoma tumor-suppressor gene (*Rb*), the von Hippel-Lindau tumor-suppressor gene (*VHL*), *p16*^*INK4a*^, the breast-cancer susceptibility gene 1 (*BRCA1*), and the MutL homolog 1 gene (*hMLH1)* [4,28,40–43]. Profiles of hypermethylation of the CpG islands in tumor-suppressor genes are specific to the cancer type so that each tumor can be assigned a specific, defining DNA “hypermethylome.” [44–46]. In acute myelogenous leukemia (AML), large-scale, genome-wide DNA methylation profiling reveals the existence of distinct DNA methylation patterns and identifies novel, biologically, and clinically relevant defined AML subgroups [47]. For example, the function of the basic leucine zipper transcription factor CCAAT/enhancer binding protein-α (C/EBPα), one of the crucial transcription factors for myeloid cell development, is frequently abrogated in AML by mutations but also through epigenetic modification through hypermethylation of the *CEBPA* promoter [48–52].

Furthermore, hypermethylation of CCCTC-binding factor (CTCF) sites has been shown to disrupt the function of insulators, which separate different genomic loops from each other [53–55]. In IDH mutated gliomas, this mechanism leads to the close interaction of *FIP1L1* gene and Platelet-Derived Growth Factor Receptor, Alpha Polypeptide (*PDGFRA)* gene, which are normally confined to separate loop domains [53]. This allows the constitutive enhancer *FIP1L1* to interact aberrantly with *PDGFRA*, a prominent glioma oncogene. This has not been so far demonstrated in hematological malignancies.

Importantly, epigenetic integrity itself can be disrupted in two different ways. Epigenetic regulators can be directly mutated, or they can be epigenetically modified, leading to a positive feedback and a drift from a tightly regulated epigenetic set point. This leads to a growth advantage of cancer cells [56]. In most solid tumors, epigenetic mutations are rather rare; they are mainly found in hematologic malignancies, rare childhood cancers, and highly aggressive solid tumors like glioblastoma multiforme [57]. Much of what we know about the epigenetic dysregulation in cancer has been elucidated by studying hematologic malignancies, because most direct epigenetic mutations (both in epigenetic writers/erasers and writers) are found in hematologic cancers.

### Mutations Involving Epigenetic Writers/Erasers

Many mutations in epigenetic regulators have been described [10,11,58–60] (see Table 1 for detailed review of mutational frequency, mechanism, and prognostic relevance of these mutations). Mutations in regulators of DNA methylation/hydoxymethylation are found in the DNA (5-cytosine)-methytransferase 3A (DNMT3A) [11,61–64] and the TET family of proteins (Fig 1) [63,65–68].

**Table 1:** Mutations in epigenetic regulators in myeloid malignancies.

Gene	Mutational frequency in myeloid malignancies	Mechanism	Impact on outcome
**Mutations in DNA modifying enzymes**			
**DNMT3A** [61]	AML: 4%–22% up to 36% (CN-AML) 16%–22% (AML > 60y) 17.8%–23% (AML < 60y) high dose: -68.3% MDS: 8% MPN: 7%–15%	DNMT3A possesses DNA methyltransferase activity, which leads to the addition of a methyl group at the 5-position of cytosine of DNA 5-methylcytosine [5mC]. *DNMT3A* mutations result either in premature truncation of the protein product (nonsense or frameshift mutations), or occur at a single amino acid, R882 (60% of mutations). In most cases, one *DNMT3A* allele remains wild-type, as haploinsufficiency seems sufficient to contribute to myeloid transformation.	Adverse risk in patients with CN-AML and FLT3-ITD mutations [62]. Improved outcome with high dose daunorubicin [63]. Single study showed adverse prognosis of *DNMT3A* mutations in MDS [64]. There is no known prognostic importance, if any, in patients with MPN [11,67].
**TET2** [65]	AML: 8%–23% 18%–23% (CN-AML) 19%–24.5% (AML > 60y) 7%–10% (AML < 60y) MDS: 20%–25% MPN: 4%-13%	TET2 possesses DNA dioxygenase activity, which leads to the conversion of the methyl group at the 5-position of cytosine of DNA 5- methylcytosine [5mC] to 5-hydroxy-methylcytosine [5hmC]. TET2 enzymes are dependent on Fe(II) and α-ketoglutarate (α-KG).	Adverse risk in patients with CN-AML independent from FLT3-ITD mutational status [63,66]; no clear prognostic importance in MDS and MPN [67,87].
**Mutations in histone modifying enzymes**			
**EZH2** [59,68,69]	AML: Rare MDS: 6%–7% MPN: 3%–13%	EZH2 is the catalytic subunit of the PcG Repressor Complex 2 (PRC2), a highly conserved Histone H3K27 methyltransferase. EZH2 mutations have a complex role, as they result both in gain and loss of function. *EZH2* may serve a dual purpose as an oncogene and tumor-suppressor gene. [69]. Biological effects of mutations unclear as EZH2 conditional knockout leads to minimal myeloid haematopoietic defects [11].	Adverse risk in all studies to date (AML, MDS, and MPD) [11,68].
**ASXL1** [11,70]	AML: 6%–30% 16.2%–25% (AML > 60y) 3%–6.8% (AML < 60y) MDS: 14% MPN: 2%–23%	Unclear whether ASXL1 mutations confer a loss or gain of function. Their role in mammalian haematopoietic-specific context is not known [74,75].	Adverse risk in patients with CN-AML, intermediate risk AML [88], and MDS [87]. Significantly associated with *RUNX1* and NPM1 mutations.
**Mutations in enzymes regulating metabolic input**			
**IDH1/2** [14,76,77,89]	5%–30% (all AML) IDH-1 (mutations at Arg132) 10%–16% (CN-AML) 9.6%–14% (AML > 60y) 7%–10.9% (AML < 60y) IDH-2 (mutations at Arg140 or Arg172) 10%–19% (CN-AML) 8%–19% (AML > 60y) 8%–12.1% (AML < 60y)	IDH converts isocitrate to α-KG, which is essential for TET2 function and mutated IDH has neomorphic enzymatic activity, which converts α-KG to 2-HG (“oncometabolite”). *IDH1/2* mutations share a mutual exclusivity with *TET2* mutations. *IDH1/2* mutations are significantly associated with *NPM1* mutations.	Conflicting studies about the prognostic relevance of IDH mutations [79].

Mutations affecting histone modification are found in the Methyltransferase Enhancer of Zeste Homologue 2 (EZH2) [59,68,69] and the additional sex combs such as 1 transcriptional regulator (ASXL1) [11,70]. Apart from playing a role in myeloid malignancies [68,69], EZH2 mutations (at codon 641) have been found to be common in follicular and diffuse large B-cell lymphomas of germinal center origin and are a promising target in these lymphomas [71–73]. The role of ASXL1 mutations in myeloid malignancies is less well understood. ASXL1 is not thought to possess enzymatic activity [74] but may be important for the recruitment of EZH2 and the stability of the PRC2 complex as demonstrated in co-immunoprecipitation experiments (Fig 1) [75].

Mutations in IDH have been discovered first in glioblastoma and then in AML (Fig 1) [14,76–78]. IDH2 mutations at the active enzyme site at position R172 and R140 confer a gain-of-function and result in a neomorphic enzymatic activity of the mutated IDH enzyme: mutant IDH1/2 catalyzes the conversion of alpha ketoglutarate to beta-hydroxyglutarate (2-HG) [79]. Supra-normal levels of intracellular 2-HG lead to competitive inhibition of α -KG dependent epigenetic regulators like TET2 and subsequently to hypermethylation of DNA as well as histones and a blockade of cellular differentiation [80,81]. Importantly, IDH and TET2 seem to be almost entirely mutually exclusive, supporting the common mechanism of action of both mutations [82].

The discovery of IDH mutations has led to the concept that “oncometabolites” like 2-HG play a major role in tumorigenesis, further underscored by the interaction of epigenetics and metabolomics in cancer.

Apart from being mutated, epigenetic writers and erasers can be aberrantly recruited by fusion proteins, which are formed by chromosomal translocation. The fusion proteins PML-RARa and AML1-ETO found in patients with t(15;17) and t(8;21) AML are the two most prominent examples. Both fusion proteins recruit multiprotein complexes including both HDACs and DNMTs to alter transcription, repress differentiation genes, and drive leukemogenesis [83–85]. Recently, the *ecotropic viral integration site 1* (*EVI1*), a DNA binding zinc-finger transcription factor, has been shown to direct a unique recurrent DNA methylation signature in AML by specifically recruiting DNMTs and HDACs to target promoters [86].

### Indirect Effects of Epigenetic Enzyme Mutations- Impact on Epigenetic Reader Domains

Rearrangement of the Histone-lysine N-methyltransferase 2A/mixed-lineage leukemia gene (*KTM2A/MLL1)* is found in approximately 5% of ALL cases and around 5% to 10% of AML cases in adults. This rearrangement results in aggressive leukemia with poor prognosis and is often refractory to conventional therapies [90,91]. Central to each of the translocations seems to be abnormal transcriptional elongation involving abnormal recruitment of histone reader proteins [5,92]. Many of the common translocation partners of MLL (including *AF9*, *ENL*, *AF4*, and *ELL*) are critical members of the super elongation complex, which contains the positive transcription elongation factor b (SEC-P-TEFb complex) (Fig 1) [93–95]. The SEC-P-TEFb complex phosphorylates RNA polymerase II, facilitating transcriptional elongation, leading to transcription of crucial oncogenes like myc and bcl-2.

The SEC-P-TEFb uses “reader domains” in the form of bromodomain and extraterminal proteins (BET proteins) in order to bind to acetylated histones on chromatin. *BET* reader proteins can be targeted by small molecule inhibitors (BETi) (Fig 1) [96–98]. In addition to the ability of MLL1 fusion proteins to recruit transcriptional machinery such as the SEC, *MLL1* rearrangement promotes gene expression by elevating local H3K79me2 levels [99,100]. The only known enzyme in mammals that catalyzes methylation of H3K79 is DOT1L (disruptor of telomeric silencing 1-like), and the MLL1 fusion proteins may directly recruit DOT1L to MLL1 fusion target loci, leading to activation of *homeobox A (HOXA*) cluster genes, which induce leukemic transformation of hematopoietic progenitors. Their high expression is a hallmark in *MLL1* rearranged leukemias [101–103]. Currently, there are several DOT1L inhibitors in development (Fig 1) [100,104,105].

## Interaction of Epigenetics with Other Hallmarks of Cancer

Recurrent genetic alterations in AML can be functionally categorized matching the hallmarks of cancer described by Hanahan and Weinberg [1,106]. Disruption in epigenetic regulation has been found to collaborate with these hallmarks in cancer development in many ways [56,107–114].

**Fig 2:**
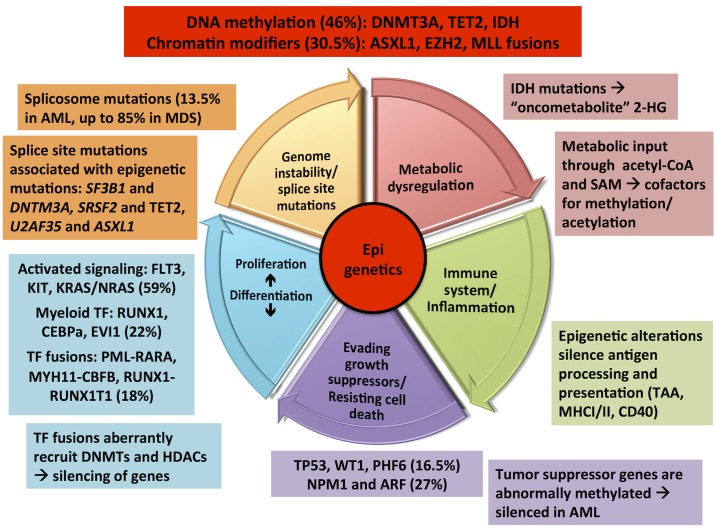
Epigenetic dysregulation as a hallmark of cancer. Overlay graphic synthesizing concepts from “Hallmarks of cancer: the next generation” by Hannahan and Weinberg [1] and “A panoramic view of acute myeloid leukemia [106]. Mutational frequency of different functional classes of mutations in parenthesis. Abbreviations: *FLT-3*: Fms-like tyrosine kinase 3; *KRAS*: Kirsten rat sarcoma viral oncogene homolog; *NRAS*: Neuroblastoma rat sarcoma viral oncogene homolog; TF: Transcription factor; RUNX1: Runt-related transcription factor 1; CEBPa: CCAAT/enhancer-binding protein alpha; *PML-RARA*: fusion of the promyelocytic leukemia (*PML*) gene on chromosome 15 to the retinoic acid receptor (*RAR*) gene on chromosome 17; *CBFB-MYH11*: chromosomal rearrangements involving the core-binding factor, beta subunit (*CBFB*) gene on chromosome 16p13.1 and the Myosin, heavy chain 11, smooth muscle (*MYH11*) gene on chromosome 16q22; *RUNX1-RUNX1T1*: chromosomal rearrangements involving the *RUNX1* gene on chromosome 21 and the Runt-related transcription factor 1 translocated to 1 (*RUNX1T1*) gene on chromosome 8. *TP53*: Tumor protein 53; *WT1*: Wilms tumor protein 1; *PHF6*: PHD Finger Protein 6; *NPM1*: Nucleophosmin 1; *ARF*: ADP-ribosylation factor 1; TAA: Tumor associated antigen; MHC I/II: Major histocompatibility complex I/II; SAM: S-Adenosyl Methionine.

Fig 2 shows an overlay of the proposed cancer hallmarks and the recurrent genetic mutations of AML with epigenetic dysregulation at the center of these complex interactions. Certain mutations seem to collaborate while others are mutually exclusive. For example, there is a strong association between mutations in the epigenetic regulator *DNMT3A*, *FLT3* (activating signaling), and *NPM1* (tumor suppressor). DNMT3A mutations may occur early in leukemogenesis and cause genetic instability, which is prone to FLT3, NPM1 mutations. On the other hand, mutual exclusivity exists among transcription factor fusion genes, *NPM1*, *RUNX1*, *TP53*, and *CEBPA*.

New research focuses on the intersection of cancer epigenetics and the newly characterized cancer hallmarks of cancer immunology [115], metabolism [116,117], and alternative m-RNA processing/splicing. Immunosuppressive microenvironment and epigenetic alterations are known to silence/downregulate all steps of antigen processing and presentation machinery (APM) in cancer cells, including tumor-associated antigens, human leukocyte antigens, and accessory/co-stimulatory molecules [118]. Epigenetic drugs have shown to up-regulate all the elements in the antigen presenting machinery, e.g., the expression of tumor associated antigens (TAA), MHC I and MHC II molecules as well as co-stimulatory surface markers like CD40, CD80 and ICAM1 [115,119–122]. There is significant evidence based on preclinical *in vitro* and *in vivo* models supporting combination therapy using epigenetic modulators and immunotherapy.

Prime examples of the interaction of the epigenome with metabolism are mutations in IDH/IDH2, which lead to accumulation of the oncometabolite 2-HG as well as acetyl CoA and S-adenosylmethionine (SAM), connecting nutritional status to gene expression through their role as donors/coenzymes for histone acetylation and DNA/histone methylation, respectively [123–125].

Mutations in splicing factors are observed in up to 85% of myeloid neoplasms with myelodysplastic features [126]. Chromatin structure and epigenetic histone modifications may act as key regulators of alternative splicing [127]. Histone marks are non-randomly distributed in the genome and are enriched specifically in exons relative to their flanking intronic regions [128]. There might be direct physical crosstalk between chromatin and the splicing machinery via an adaptor complex [129]. Importantly, each splice gene mutation seems to be associated with one concomitant mutation in a gene involved in epigenetic regulation of transcription. *SF3B1*, *SRSF2* /*ZRSR2*, and *U2AF35* mutations are enriched in patients with *DNMT3A*, *TET2*, and *ASXL1*, respectively [130]. On the other hand, mutations in the splicing factor genes U2AF1 and SRSF2 cause dysfunctional processing of pre-mRNA and reduced EZH2 expression [69].

The cohesin complex is important in mediating proper sister chromatid cohesion and separation from S phase to M phase in mitosis as well as in regulating transcription through genome-wide chromatin organization. Mutations of proteins of the complex are frequently found in myeloid neoplasms [131]; they collectively occur in approximately 15% of AML cases and other myeloid malignancies [132]. Leukemia-associated cohesion mutations have been found to impair differentiation and enforce stem cell programs in human stem and progenitor cells by demonstrating increased chromatin accessibility of stem cell regulators like the Runt-related transcription factor 1 (RUNX1) and GATA2 [133,134].

## Epigenetic Therapy

### Current Therapies

To date, epigenetic therapies have been limited to targeting epigenetic writers in the form of DNA methyltransferase inhibitors (DNMTi) and epigenetic erasers in the form of histone deacetylase inhibitors (HDACi) (Fig 1). The Food and Drug Administation (FDA) approved two DNMTi (azacitidine and decitabine) for the treatment of MDS [135] and several HDACi (vorinostat, romidepsin, belinostat, and panobinostat) for the treatment of cutaneous T-cell lymphoma and multiple myeloma [136–140], respectively. Due to their pleitropic effects, it has been difficult to confirm the mechanism of action of DNMTi and HDACi.

## Emerging Future Therapies

### Isoform Specific HDAC Inhibitors

Current research focuses on developing specific therapy by using isoform-specific HDACi [141]. For example, the class I HDAC inhibitor entinostat was recently awarded by the FDA a breakthrough therapy status for patients with metastatic, estrogen receptor-positive breast cancer based on data from the phase II ENCORE 301 study (NCT00676663) [142].

### Novel Epigenetic-Targeted Pharmacologic Agents

Several new agents targeting epigenetic writers and erasers are in development, including EZH2 inhibitors [73,143], protein methyltransferase inhibitors (PMT inhibitors) [144], and histone lysine demethylases (KDM inhibitors) [145]. Several phase I/II clinical trials will be dedicated to studying the effect of these new agents in patients (see Table 2).

**Table 2:** Selection of ongoing clinical trials evaluating epigenetic targeted therapies in hematologic malignancies.

Clinical Trial	Intervention	Malignancy studied
**EZH2 inhibitors**		
**NCT02395601**	Phase 1 Study: EZH2 inhibitor CPI-1205	Progressive B-cell lymphomas
**NCT01897571**	Phase 1/2 Study: EZH2 inhibitor E7438	B-cell lymphomas and advanced solid tumors
**KDM inhibitors**		
**NCT02261779**	Phase 1/2 Study: ATRA + tranylcypromine (TCP) an irreversible monoamine-oxidase (MAO) and Lysin-specific demethylase (LSD) inhibitor	Relapsed/refractory AML
**IDH2 inhibitors**		
**NCT01915498**	Phase 1/2 Study: reversible inhibitor of mutant IDH2 AG-221	Advanced hematologic malignancies with IDH2 mutation
**NCT02273739**	Phase 1/2 Study: reversible inhibitor of mutant IDH2 AG-221	Advanced solid tumors (glioma) and angioimmunoblastic T-cell lymphoma
**BET1/DOT1L inhibitors**		
**NCT01943851**	Phase 1/2 Study: BET inhibitor GSK525762	Relapsed/refractory hematologic malignancies (leukemias, myeloproliferative neoplasms, lymphomas, and myelomas)
**NCT02158858**	Phase 1 Study: BET inhibitor CPI-0610	AML, myelodysplastic syndromse, myeloproliferative neoplasms, myelofibrosis
**NCT02308761**	Phase 1 Study: BET inhibitor TEN-010	AML, myelodysplastic syndromse
**NCT01684150**	Phase 1 Study: second generation DOT1L inhibitor EPZ-5676	AML/ALL/MLL with *MLL1* rearrangements (including 11q23 or partial tandem duplications) in adult patients
**NCT02141828**	Phase 1 Study: second generation DOT1L inhibitor EPZ-5676	AML/ALL with *MLL1* rearrangements (including 11q23 or partial tandem duplications) in pediatric patients
**Combination treatment with cancer vaccines**		
**NCT01483274**	Phase 1 study: Decitabine + donor lymphocyte infusion + Vaccine (autologous dendritic cells)	AML with relapse after allogeneic stem cell transplantation
**Combination treatment with immune checkpoint inhibitors**		
**NCT02281084**	Phase 2 Study: Durvalumab (PD-L1 inhibitor) + CC-486 (oral azacitidine)	Myelodysplastic syndromes
**NCT02530463**	Phase 2 Study: Nivolumab (PD-1 inhibitor) and/or Ipilimumab (CTLA-4 inhibitor) + azacitidine	Myelodysplastic syndromes

This list is not complete but presents a selection of clinical trials by the authors of this manuscript meant to illustrate the different strategies.

IDH inhibitors seem to be particularly promising [77,89,144]. Early results of AG-221, an inhibitor of mutant IDH2, showed that from 48 patients with advanced AML/MDS with an IDH2 mutation, 20 patients had evidence of an objective response (eight complete remissions) (see Table 2) [146,147]. Other compounds like pan IDH and IDH1 inhibitors (AG-120) are in development.

Exciting new data also comes from drugs developed to target leukemias harboring *MLL1* translocations, BET inhibitors, and DOT1L inhibitors (see Table 2) [5,100,104]. The MLL fusion protein can aberrantly recruit multiprotein complexes including SEC and DOT1L, activating important oncogenic genes like HOXA cluster genes, c-myc, bcl-2, and others, but can be interrupted by targeting the reader proteins BET within SEC or DOT1L directly. Interestingly, BET inhibitors and DOT1L inhibitors are also effective in vitro in a variety of other leukemias [148] and hematologic malignancies such as multiple myeloma or Burkitt lymphoma [149,150], for which *HOXA* or *c-myc* activation are key drivers of the disease.

## Combination Strategies

In combining epigenetic agents with cytotoxic chemotherapy, the reactivation of tumor-suppressor genes and restoration of DNA-repair pathways by epigenetic drugs results in more chemo-sensitive cells. These cells can then be targeted by another type of therapy [151]. Initial studies combining epigenetic agents with chemotherapy showed disappointing results [152], though further studies suggest that the timing of epigenetic therapy matters and that it might be able to reverse resistance to chemotherapy [153–156].

Initial approaches focused on combining epigenetic agents with cytokine-based immunotherapy and vaccination with tumor cells or peptide vaccines [118,157–159]. With the dawn of the checkpoint inhibitors CTLA-4 and PD-1/ PDL-1 to stimulate the immune system in solid malignancies, the combination of immune checkpoint inhibitors with epigenetic therapy has been promising in preclinical models [160–162]. Several ongoing phase-I/II clinical trials are dedicated to investigating the effect of combining epigenetic agents with immunotherapy (see Table 2) [115,161].

## Discussion/Conclusion

We have come a long way in understanding the epigenetic network from the initial model of epigenetic regulation: DNA gets methylated, recruits histone deacetylases, and the two systems button down chromatin and silence expression. With the availability of NSG, CHIP-Seq, and Crisp-cas9 technologies, we now understand that epigenetics involves a complex and dynamic interplay of writers, erasers, and readers, which act not only on promoters but on many regulatory elements, including enhancers and repressors, forming a three-dimensional network of regulation.

The impact of epigenetic alterations in cancer is also complex. Many mutations in epigenetic writers, erasers, and readers have been identified as promoting cancer development. Furthermore, epigenetics has been recognized as lying at the heart of multiple hallmarks of cancer, interacting with cell cycle promotion, cancer metabolism, neo angiogenesis and the immune system. Although not able to induce leukemia [163,164], a dysregulated epigenome allows other mutations to occur, giving cancer cells a growth advantage over normal cells. Unsurprisingly, epigenetic mutations are starting to get used as biomarkers in hematologic malignancies and have been found to be associated with poor prognosis.

The parallel development of epigenetic therapy mirrors the evolution of biology. The “historical” DNMTi and HDACi are registered in hematological cancers, but their mode of action is not fully understood, while more recently, NGS-driven drug discovery has led to the development of real targeted agents focused on epigenetic writers and IDH. Several important questions have to be answered: What is the optimal duration of therapy with epigenetic agents? Several of these agents might require longer exposure to have a therapeutic effect compared to traditional cytotoxic chemotherapy and small molecule tyrosine kinase inhibitors [58,73,104,143]. Second, can we develop biomarkers to predict response to epigenetic therapy? CpG island methylation signatures have only been mildly successful in predicting therapy response [56,165,166]. Furthermore, as is the case for the majority of tumors sensitive to BET and DOT1L inhibition, epigenetic therapeutic targets are not necessarily mutated in sensitive tumor types. And although a central theme of BET inhibition seems to be c-myc downregulation, there are multiple cancer cell lines that overexpress c-myc but do not respond to BETi [5,150], as c-myc downregulation does not predict a response [96,98]. Therefore, simple mutational screening or gene expression profiling may not provide a predictor of response and might require large drug screening studies to test sensitivities [167,168]. Last, but not least, will there be a role for chemoprevention similar to using statins in heart disease? As epigenetics is recognized as a very early driver for cancer progression, this holds promise for both improved early diagnosis and therapy of cancer [56]. Will it be possible in the future to identify patients early in the course of their disease and treat even before development of overt cancer based on their epigenetic profile? There is some evidence that this might be possible in colorectal and cervical cancer [169,170]. As single mutations in epigenetic regulators cannot induce cancer on their own, single epigenetic agents will only be part of the cure. AML and cancer in general is a multi-step process, and targeting a single defect (as has been seen with Flt-3 inhibitors) is not sufficient to control cancer. In that context, one of the most promising approaches is using epigenetic therapy in combination with other therapies targeting different hallmarks of cancer, including traditional chemo- and radiation therapy as well as immunotherapy, which is currently changing the paradigm of therapy in solid malignancies [171]. Although most data are generated in vitro and in mouse models, there is evidence that the use of epigenetic drugs improves the antitumor activity of immune checkpoint inhibitors. As the overall effectiveness of immunotherapy is still far from optimal—only a minority of treated patients achieve long-term clinical benefit [172]—and there are poorly immunogenic tumors like AML, epigenetic therapy could serve as an essential part of future combination immune therapy [118]. There might be two sides to the coin in terms of pleiotropic effects of epigenetic agents: initially viewed as a weakness, it might prove to be an advantage in the light of combination therapy.

Understanding the impact that epigenetics has on cancer biology, diagnosis, and therapy is complex and fascinating and holds great promise for the future.
